# Diabetic Retinopathy (DR): Mechanisms, Current Therapies, and Emerging Strategies

**DOI:** 10.3390/cells14050376

**Published:** 2025-03-04

**Authors:** Hyewon Seo, Sun-Ji Park, Minsoo Song

**Affiliations:** New Drug Development Center, Daegu-Gyeongbukk Medical Innovation Foundation (K-MEDI hub), 80 Cheombok-ro, Dong-gu, Daegu 41061, Republic of Korea; h.seo@kmedihub.re.kr (H.S.); sunji@kmedihub.re.kr (S.-J.P.)

**Keywords:** diabetic retinopathy, molecular mechanism, therapeutics, oxidative stress, inflammation, diabetic complications

## Abstract

Diabetic retinopathy (DR) is one of the most prevalent complications of diabetes, affecting nearly one-third of patients with diabetes mellitus and remaining a leading cause of blindness worldwide. Among the various diabetes-induced complications, DR is of particular importance due to its direct impact on vision and the irreversible damage to the retina. DR is characterized by multiple pathological processes, primarily a hyperglycemia-induced inflammatory response and oxidative stress. Current gold standard therapies, such as anti-VEGF injections and photocoagulation, have shown efficacy in slowing disease progression. However, challenges such as drug resistance, partial therapeutic responses, and the reliance on direct eye injections—which often result in low patient compliance—remain unresolved. This review provides a comprehensive overview of the underlying molecular mechanisms in DR, the current therapies, and their unmet needs for DR treatment. Additionally, emerging therapeutic strategies for improving DR treatment outcomes are discussed.

## 1. Introduction

Diabetic retinopathy (DR) is one of the most prevalent microvascular complications of diabetes mellitus and a leading cause of vision loss across all age groups, including children, working adults, and the elderly [1,2,3]. In 2020, more than 103 million people worldwide were affected by DR, and projections estimate this number will rise to 161 million by 2045 [4]. Clinically, DR is categorized into two stages: non-proliferative DR (NPDR), characterized by vascular abnormalities, and proliferative DR (PDR), which involves pathological neovascularization. Severe NPDR increases the risk of progression to PDR, while diabetic macular edema (DME)—a major cause of vision impairment—can develop at any stage of DR.

The burden of DR extends beyond vision loss, encompassing significant physical, emotional, and socioeconomic challenges. Although early-stage DR is often asymptomatic, early diagnosis plays a crucial role in improving treatment outcomes. However, current diagnostic methods, such as optical coherence tomography (OCT), fluorescein angiography (FA), and color fundus retinal photography, require in-person clinical visits, which may pose accessibility challenges for individuals in remote areas [5], and those from low-income households [6,7]. To enhance early detection and risk assessment, recent research has explored genetic prediction analysis [8,9], patient history evaluation [10], biomarker discovery [11], and artificial intelligence (AI)-based diagnostic tools that analyze DR-related retinal features from imaging data [12,13]. Despite these advancements, effective treatment options remain an unmet clinical need.

Currently, DR is managed through intravitreal injections of anti-vascular endothelial growth factor (anti-VEGF) drugs, vitreous surgery, and laser therapy. Anti-VEGF agents, such as bevacizumab, ranibizumab, and aflibercept, have demonstrated clinical efficacy in reducing vascular leakage and neovascularization. However, only 29% of patients with DME experience significant visual improvement after two years of treatment [14]. Moreover, the need for frequent intravitreal injections and the high cost of antibody-based therapies impose substantial financial and logistical burdens on patients. While strict glycemic control has been shown to slow the onset and progression of DR [15,16,17], some studies indicate that DR may continue to progress even in patients with intensive glycemic management due to the phenomenon of “metabolic memory”—a process that remains poorly understood [18].

Given the limited efficacy of current therapies and the rising global prevalence of DR, there is an urgent need for novel therapeutic approaches. This review provides a comprehensive overview of DR, focusing on current treatment modalities, existing challenges, and emerging strategies aimed at improving patient outcomes.

## 2. Molecular Mechanism of DR

**Figure 1:**
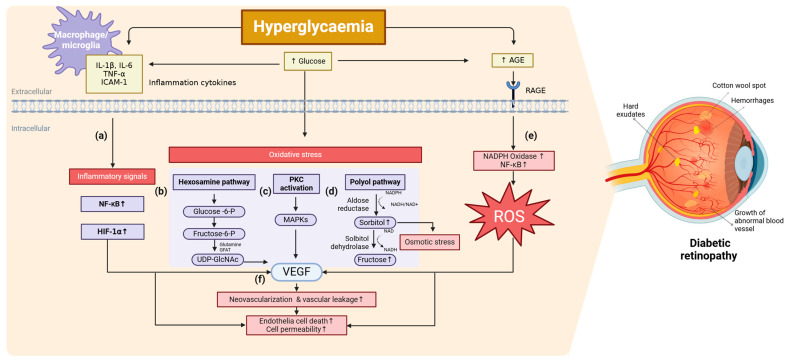
Mechanisms of hyperglycemia-induced pathogenesis in DR. Elevated glucose levels induce inflammatory responses, oxidative stress, and metabolic dysregulation, which lead to endothelial dysfunction, vascular leakage, and neovascularization in the retina. Hyperglycemia activates macrophages and microglia, promoting the release of pro-inflammatory cytokines (IL-1β, IL-6, TNF-α, ICAM-1), which further stimulate NF-κB and HIF-1α signaling (**a**). Oxidative stress enhances the activation of metabolic pathways, including the hexosamine pathway (**b**), protein kinase C (PKC) activation (**c**), and the polyol pathway (**d**), contributing to VEGF upregulation and vascular dysfunction. Additionally, hyperglycemia leads to the formation of advanced glycation end-products (AGEs), which activate RAGE signaling (**e**), resulting in increased NADPH oxidase activity and reactive oxygen species (ROS) production. The accumulation of ROS exacerbates oxidative stress, endothelial cell death, and vascular permeability. The cumulative effect of these processes results in pathological neovascularization and vascular leakage (**f**), leading to retinal damage characteristic of DR. The right panel depicts retinal abnormalities and typical fundus features associated with DR, including increased neovascularization, cotton wool spots, and hard exudates. Created in BioRender. Seo, H. (2025) https://BioRender.com/n23b627 (accessed on 30 January 2025).

Extensive research has investigated the biochemical and molecular mechanisms underlying the onset and progression of DR; however, no single definitive pathway has been established. Key pathological features of DR include pericyte loss, thickening of the basement membrane, microaneurysm formation, neovascularization, and disruption of the blood–retinal barrier. Hyperglycemia-induced oxidative stress, inflammation, increased glycosylation products, and vascular endothelial growth factor (VEGF) overexpression have all been identified as critical contributors to DR pathogenesis. These mechanisms are highly interconnected, forming a complex network of pathological events (Figure 1).

### 2.1. Inflammation

Inflammation plays a key role in DR progression, with several pro-inflammatory cytokines—such as interleukin 1β (IL-1β), IL-18, and tumor necrosis factor-α (TNF-α)—being significantly upregulated and implicated in inflammation-induced cell death (Figure 1a).

Excessive IL-1β triggers apoptosis in retinal pigment epithelial cells, compromising photoreceptor integrity [19]. Additionally, IL-1β activates the nuclear factor kappa B (NF-κB) pathway and oxidative stress, leading to endothelial cell apoptosis and increased vascular permeability [20]. Emerging studies indicate that IL-17A contributes to PDR progression. Müller cells, which constitute 90% of the glial cells in the retina and mediate intercellular communication, transmit IL-17A signals to retinal ganglion cells via exosomal miRNA (miR-92a-3p), leading to Notch-1 downregulation. This finding suggests that targeting miR-92a-3p could be a potential therapeutic approach [21].

Macrophage and microglia polarization further contribute to the pathological progression of DR. These immune cells are key regulators of innate immunity, orchestrating various homeostatic and host defense responses [22]. In response to environmental signals, macrophages/microglia can polarize into two distinct yet reversible phenotypes: M1 and M2. M1 macrophages are pro-inflammatory, releasing cytokines that exacerbate inflammation, while M2 macrophages contribute to tissue repair, remodeling, and the clearance of necrotic cell debris [23]. Recent research suggests that macrophage polarization extends beyond the binary M1/M2 classification, with intermediate or hybrid phenotypes—such as tumor-associated macrophages—exhibiting characteristics of both, along with other macrophages that do not fit within this classification, indicating a broader spectrum of functional states [24,25]. Under hyperglycemia conditions, M1 macrophage activation is heightened, leading to increased production of pro-inflammatory factors, while M2 macrophage activity and anti-inflammatory factor production are diminished [26]. Studies have proposed that enhancing M2 polarization could be a potential therapeutic strategy for diabetes by reducing insulin resistance and stabilizing glucose and lipid metabolism [27]. More recently, Liu et al. identified a link between mitochondrial dysfunction and macrophage polarization in DR. As mitochondria serve as the primary source of ATP for macrophages, their function is crucial in regulating polarization. By analyzing 784 differentially expressed genes of the GSE221521 dataset, researchers identified intersections between mitochondrial-related genes (MRGs) and macrophage polarization-related genes (MPRGs) in patients with DR. Mendelian randomization (MR) analysis further pinpointed 13 genes with strong causal relationships, and machine learning algorithms identified protein prenyltransferase alpha subunit repeat-containing protein 1 (PTAR1) and SLC25A34 as potential biomarker genes associated with DR pathology [28].

VEGF is another key inflammatory factor driving neovascularization and vascular leakage in DR (Figure 1f) [29]. Under hyperglycemic and hypoxic conditions, hypoxia-inducible factor-1α (HIF-1α) is activated, leading to increased VEGF secretion. VEGF promotes neovascularization through signaling pathways such as PI3K/Akt, PKC, and NF-κB [30]. Furthermore, VEGF enhances the expression of intercellular adhesion molecule-1 (ICAM-1) and nitric oxide synthase, facilitating leukocyte adhesion, vascular permeability alterations, and pathological neovascularization.

### 2.2. Oxidative Stress

Chronic hyperglycemia induces oxidative stress through multiple pathways, including the polyol pathway, hexosamine biosynthesis, advanced glycation end-product (AGEs) formation, and protein kinase C (PKC) activation.

The hexosamine pathway produces uridine-5′-diphospho-N-acetylglucosamine (UDP-GlcNAc), a key substrate for N-linked glycosylation of secretory proteins and O-GlcNAcylation of intracellular proteins at serine/threonine residues. Glycosylation influences protein activity, stability, and function. It begins with the conversion of glucose into fructose-6-phosphate, which is subsequently transformed into glucosamine-6-phosphate by glutamine-fructose-6-phosphate amidotransferase (GFAT), the rate-limiting enzyme in this pathway (Figure 1b). Overexpression of GFAT has been reported in diabetic glomeruli, correlating with postprandial hyperglycemia and insulin resistance [31]. Moreover, increased O-GlcNAcylation in diabetic models disrupts gene expression by modifying transcription factor Sp1, leading to VEGF upregulation [32,33]. Furthermore, O-GlcNAcylation of p53 has been linked to retinal pericyte apoptosis, contributing to early vascular dysfunction in DR [34,35].

Hyperglycemia-induced PKC activation, particularly PKC-β, is implicated in endothelial dysfunction, increased vascular permeability, and excessive neovascularization in DR (Figure 1c) [36]. PKC activation is driven by elevated diacylglycerol (DAG) levels, which trigger signaling cascades that involve mitogen-activated protein kinases (MAPKs), transcription factors, and oxidative stress pathways. This leads to the overexpression of plasminogen activator inhibitor-1, VEGF, and other pro-inflammatory and pro-angiogenic factors. While PKC inhibitors have potential therapeutic applications, further studies are required to validate their clinical efficacy.

The polyol pathway converts glucose to sorbitol via aldose reductase and subsequently metabolizes it into fructose (Figure 1d). This conversion consumes NADPH, thereby depleting cellular antioxidant defenses and increasing the NADH/NAD^+^ ratio, which contributes to oxidative stress and osmotic imbalance. Aldose reductase, a key enzyme in this pathway, has low glucose affinity but becomes hyperactive under hyperglycemic conditions, leading to sorbitol accumulation in the retina, kidneys, and nerves [37]. Since sorbitol diffuses poorly across cell membranes, it exacerbates osmotic stress and cellular damage [38]. While aldose reductase inhibitors showed early promise in animal models, clinical trials, such as the sorbinil trial yielded inconsistent results, casting doubt on the therapeutic feasibility of targeting this [39].

Prolonged hyperglycemia results in the formation of AGEs, which accumulate in retinal vessels and exacerbate DR pathology [40]. AGEs, generated through the non-enzymatic glycation and oxidation of proteins and lipids, contribute to vascular damage by binding to the receptor for advanced glycation end-product (RAGE). This interaction activates inflammatory and oxidative stress pathways, including NF-κB and MAPK, further amplifying retinal damage (Figure 1e). AGEs, such as carboxyethyllysine, carboxymethyllysine, and pentosidine, are highly enriched in the retinal tissues of patients with DR. Although AGEs are established contributors to DR pathogenesis, further research is needed to better understand their mechanisms and potential as a therapeutic target.

### 2.3. Other Related Pathways

Recently, Jeon et al. have identified the transglutaminase 2 (TGase2)/AMP-activated protein kinase (AMPK)/ glyceraldehyde 3-phosphate dehydrogenase (GAPDH) pathway as a key molecular mechanism underlying hyperglycemia-induced microvascular leakage in DR [41]. This pathway is activated by elevated intracellular calcium (Ca^2+^) and reactive oxygen species (ROS), which cause activated TGase2 to inhibit AMPK phosphorylation and GAPDH activity. This leads to the disassembly of adherens junctions and vascular permeability in the retina. Since AMPK plays a protective role in maintaining the blood–retinal barrier and reducing inflammation, its suppression by activated TGase2 accelerates DR progression. Additionally, GAPDH inhibition contributes to vascular dysfunction by exacerbating oxidative stress and increasing endothelial permeability. This pathway offers potential therapeutic targets for managing DR and related diabetic vascular complications.

An interesting study has examined why some patients with NPDR do not progress to PDR. MicroRNA sequencing of plasma and retinal samples from patients with NPDR who did not develop PDR revealed that the level of circulating microRNAs targeting ETS proto-oncogene 1 (Ets1), a crucial regulator of microvascular angiogenesis, is highly upregulated. Further analysis using single-cell sequencing data showed that Ets1 expression was reduced in diabetic endothelial cells, which was associated with the suppression of Angiopoietin-1 and the PI3K-Akt signaling pathways. These findings suggest that an anti-angiogenic mechanism may be active in NPDR, preventing progression to the more severe form of the disease, PDR [42].

## 3. Current Clinical Therapies

For patients with mild to moderate NPDR, regular screening and blood glucose monitoring are recommended [43]. However, for patients with PDR and DME, active therapeutic interventions are essential to prevent vision loss. Current treatments for PDR and DME primarily include laser photocoagulation, intravitreal injections of anti-VEGF or steroids, and vitreoretinal surgery. A list of currently approved drugs for DR treatment is provided in Table 1.

Laser therapy, particularly panretinal photocoagulation (PRP), has long been a cornerstone in the management of DR. Clinical trials have demonstrated that PRP significantly reduces the risk of progression to high-risk PDR and decreases the likelihood of severe vision loss by over 50% [44]. PRP works by creating laser-induced burns in the peripheral retina, leading to tissue coagulation and improved retinal oxygenation [45]. However, PRP treatment is not a one-time solution. As shown in RIDE (ranibizumab injection in subjects with clinically significant macular edema with center involvement secondary to diabetes mellitus) and RISE (ranibizumab injection in subjects with clinically significant macular edema with center involvement secondary to diabetes mellitus) clinical trials, 9.5% of patients required multiple PRP treatment over 24 months without additional anti-VEGF treatment [46]. While PRP effectively reduces vision loss, it is associated with significant side effects, including peripheral vision loss, delayed dark adaptation, and creeping atrophy [47,48,49].

Another effective treatment is intravitreal anti-VEGF injection. Initially developed for DME, ranibizumab (Lucentis; Genentech, Inc., South San Francisco, CA, USA), bevacizumab (Avastin; Genentech, Inc., South San Francisco, CA, USA), and aflibercept (Eylea; Regeneron Pharmaceuticals, Inc., Tarrytown, NY, USA) have also demonstrated efficacy in treating severe DR. The Protocol S study revealed that ranibizumab treatment reduced DME, minimized visual field defects, and decreased the need for vitrectomy compared to PRP [50]. Similarly, the RIDE study showed that ranibizumab therapy led to better visual acuity and prevention of neovascularization compared to PRP after 12 months; however, these benefits diminished at 24 months. Despite these advantages, frequent follow-ups and the risk of disease worsening upon treatment discontinuation remain significant concerns.

Potential adverse effects of anti-VEGF injections include increased intraocular pressure, retinal detachment, endophthalmitis, cataract formation, and systemic absorption. Furthermore, some patients with DME exhibit incomplete responses to anti-VEGF therapy, underscoring the need for alternative, long-lasting, and non-invasive treatment options [51].

Corticosteroids (CSs) are widely used for their anti-inflammatory and immunosuppressive properties in treating inflammatory, allergic, and autoimmune conditions such as asthma, rheumatoid arthritis, and lupus. However, long-term corticosteroid use is associated with significant systemic and ocular side effects, including hypertension, diabetes, gastrointestinal complications, osteoporosis, ocular hypertension, glaucoma, cataracts, and central serous chorioretinopathy. Steroid-induced glaucoma (SIG) can lead to optic disk and vision loss if left untreated, while prolonged systemic corticosteroid use increases the risk of posterior subcapsular cataracts [52]. These potential complications highlight the need for careful patient selection and monitoring when considering corticosteroids as a treatment for DR.

Ripasudil, a Rho-associated kinase (ROCK) inhibitor, initially developed for glaucoma and ocular hypertension, has been explored for DR treatment. Aging is associated with increased ROCK2 signaling, which leads to the overexpression of proangiogenic macrophages that promote IL-4 production and angiogenesis. Ripasudil suppresses multiple inflammatory and macrophage activation markers, including NLR family pyrin domain containing 3 (NLRP3), apoptosis-associated speck-like protein containing a caspase recruitment domain (ASC), caspase-1, IL-1β, and IL-18 [53].

Non-steroidal anti-inflammatory drugs (NSAIDs) function by inhibiting cyclooxygenase (COX) enzymes, thereby reducing pro-inflammatory prostaglandin levels. Studies suggest that combining bromfenac with anti-VEGF therapy may improve visual acuity in age-related macular degeneration (AMD) [54]. In DR, NSAIDs have been proposed to lower COX and prostaglandin levels, which are often elevated in retinal cells [55]. However, the Protocol R study from the Diabetic Retinopathy Clinical Research (DRCR) network found no significant impact of NSAIDs on retinal thickness in non-central DME [56]. Moreover, concerns regarding corneal complications persist, especially in patients with diabetes or those undergoing post-surgical recovery.

Population-based studies have indicated that elevated total cholesterol, triglycerides, and low-density lipoprotein (LDL) cholesterol increase the risk of retinal hard exudates and macular edema [57,58,59,60]. However, the impact of dyslipidemia on PDR progression remains inconsistent. For example, a study in male patients with diabetes found no significant correlation between statin use and DR progression [61]. On the other hand, fenofibrate, a peroxisome proliferator-activated receptor alpha (PPARα) agonist, has shown promising results in reducing DR progression by lowering triglycerides and LDL cholesterol while increasing high-density lipoprotein (HDL) cholesterol. Two large randomized clinical trials have confirmed its efficacy in slowing DR progression [62,63].

Surgical intervention, such as vitrectomy, is reserved for severe cases, including dense vitreous hemorrhage, macula-threatening tractional detachment, or rhegmatogenous retinal detachment. Nonetheless, complications—such as recurrent vitreous hemorrhage, cataract formation in phakic patients, and, in rare instances, postoperative endophthalmitis—may occur [64,65,66]. These risks underscore the need for careful management and individualized treatment planning in DR.

## 4. Emerging Therapeutics

Emerging research is paving the way for a variety of new approaches, including small molecule inhibitors including natural compounds, and dietary interventions targeting underlying mechanisms such as inflammation, oxidative stress, and neovascularization. Meanwhile, advances in molecular biology and biochemistry have led to the identification of promising biomarkers and therapeutic targets. This section highlights recent developments in pharmacological treatments, dietary interventions, and novel therapeutic targets.

### 4.1. Natural Compounds and Supplements

Resveratrol (RES), a natural antioxidant, has been investigated for DR treatment both in vitro and in vivo. Peng et al. reported that 20 μM RES significantly improved cell viability, reduced apoptosis, decreased reactive oxygen species generation, and lowered inflammatory cytokines in high-glucose-induced human retinal capillary endothelial cells (HRCECs) [67]. Further studies on its molecular mechanism revealed that RES activates the sirtuin 1 (SIRT1)/ high-mobility group box 1 (HMGB1) pathway, as demonstrated by increased SIRT1 and HMGB1 expression and reduced HMGB1 acetylation. Silencing SIRT1 with lentiviral shRNA reversed RES’s protective effects, confirming the SIRT1/HMGB1 pathway as a critical mechanism in RES-mediated DR protection. Additionally, RES suppressed ferroptosis by restoring the levels of solute carrier family 7 member 11 (SLC7A11) and reducing iron and malondialdehyde concentrations.

In a streptozotocin (STZ)-induced DR mouse model, daily administration of 20 mg/kg RES for four weeks resulted in lower blood glucose levels, preserved retinal structure as observed through optical coherence tomography (OCT), and improved retinal function measured by electroretinography. Histological analysis further demonstrated reduced expression of VEGF and CD31, markers of angiogenesis, along with attenuation of retinal thinning and neovascularization. These effects were reversed upon SIRT1 silencing, underscoring its essential role in RES-mediated retinal protection. Given its efficacy and safety profile, RES warrants further clinical investigation as a potential therapy for DR.

Another natural compound with promising anti-inflammatory properties is oridonin (Ori), the major active component of the traditional Chinese medicinal herb Rabdosia rubescens [68]. In an STZ-induced diabetic mouse model, intraperitoneal injection of Ori improved visual function and mitigated retinal abnormalities, including yellowish-white spots, contiguous patches, and lesions resembling geographic damage observed in fundus photography. Additionally, it significantly lowered systemic levels of TNF-α and IL-1β while suppressing retinal NLRP3 inflammasome activation. The molecular mechanism studies revealed that Ori alleviated high-glucose-induced pyroptosis in human retinal endothelial cells (HRECs) by inhibiting the NF-κB/NLRP3 inflammasome pathway and disrupting NEK7–NLRP3 interactions. These findings suggest that Ori and other NLRP3 inhibitors could serve as an effective therapy for mitigating DR progression by targeting inflammation and pyroptosis.

Dietary interventions have also been explored as potential approaches for DR management. Among omega-3 fatty acids, docosahexaenoic acid (DHA), the most abundant fatty acid in the human retina, plays a crucial role in retinal function and visual acuity [69]. In both patients with diabetes and mouse models, DHA levels in the retina were significantly reduced. Postmortem analyses revealed a 40% reduction in DHA in the peripheral retinas and a 25% decrease in the macula of diabetic human donors [70]. Similar reductions were observed in diabetic mice, along with thinner retinal layers and impaired retinal function. Gene expression analysis indicated increased pro-inflammatory TNF-α levels and downregulation of elongation of very-long-chain fatty acid protein 5 (ELOV5), an enzyme essential for DHA biosynthesis. These results indicate that diabetes-induced hyperglycemia and inflammation contribute to oxidative stress, impaired DHA synthesis, and reduced DHA uptake, ultimately leading to structural and functional retinal impairments that may accelerate DR progression. Therefore, maintaining adequate DHA levels through dietary supplementation has been proposed as a potential strategy to prevent or slow the progression of DR. For example, high doses of DHA (1 g) combined with carotenoids have been shown to significantly improve macular function within three months in asymptomatic patients with NPDR [71]. And the combination of dietary DHA with overexpression of Mfsd2a, the lysophosphatidylcholine-DHA transporter, also showed a reduction in neovascularization in both STZ- and oxygen-induced diabetic mouse models [72]. Although further studies are needed to establish a causal relationship between DHA depletion and DR progression, these findings suggest that dietary DHA enrichment could be a new approach to preventing the progression of early-stage DR.

### 4.2. Lipid Modulators

Lipid metabolism has been increasingly recognized as a contributing factor in DR pathology. Proprotein convertase subtilisin/kexin type 9 inhibitors (PCSK9i), including alirocumab, evolocumab, and inclisiran, are widely used to reduce LDL-C levels and lower cardiovascular risks in patients with diabetes. Recent studies suggest that PCSK9 inhibitors could offer additional therapeutic benefits in DR. Serum PCSK9 levels have been positively correlated with advanced DR stages, indicating a possible role in disease progression [73]. Notably, evolocumab has demonstrated anti-inflammatory effects in retinal Müller cells by modulating the TLR-4/NF-κB signaling pathway [74]. A MR analysis by Chen et al. further identified a genetic association between PCSK9-mediated LDL-C regulation and DR risk, supporting the idea that PCSK9 inhibitors could be repurposed for DR treatment [75]. Clinical trials have also provided evidence for the role of lipid-lowering drugs in DR management. In the multinational FIELD study, which included 9795 patients with type 2 diabetes, daily administration of fenofibrate (200 mg) significantly reduced the need for laser treatment in patients with DR [76]. Similarly, the ACCORD-EYE study reported a 3.7% reduction in DR progression over four years in patients with pre-existing DR who were treated with fenofibrate [77]. These findings highlight the potential of lipid-lowering therapies, particularly PCSK9 inhibitors and PPARα agonists, in improving DR outcomes.

### 4.3. Epigenetic Modulators

Epigenetic modifications have been increasingly implicated in DR pathogenesis, particularly in inflammation-mediated retinal damage. Histone deacetylase 6 (HDAC6) has been identified as a key regulator of NLRP3 inflammasome activation, driving retinal inflammation and neurodegeneration in DR [78]. RNA sequencing of retinal tissue from STZ-induced diabetic mice fed a high-fat diet revealed a significant upregulation of HDAC6 [79]. Notably, HDAC6 knockout (KO) mice exhibited marked improvement in DR symptoms such as reduced retinal vascular leakage and preserved retinal structure, without affecting blood glucose levels. Furthermore, oral administration of compound **1** (4-((4-(1-(2-fluoro-2-methylpropyl)piperidin-4-yl)-1H-pyrrolo[2,3-b]pyridin-1-yl)methyl)-N-hydroxybenzamide), developed by Chong Kun Dang, at a dose of 50 mg/kg daily for one week, led to decreased vascular leakage, reduced inflammatory markers, and improved retinal integrity in diabetic mice. Further insights were obtained from both in vivo and in vitro studies; notably, bone marrow-derived macrophages exposed to AGEs showed that HDAC6 KO macrophages produced lower levels of IL-1β and TNF-α, confirming that HDAC6 plays a key role in the inflammatory response under diabetic stress. Collectively, these findings highlight the therapeutic potential of selective HDAC6 inhibition as a novel strategy for treating DR and other diabetic complications.

### 4.4. Other Targeted Therapies

Huang et al. reported that peroxiredoxin 4 (PRDX4), a protein that removes hydrogen peroxide and helps protein folding in the endoplasmic reticulum (ER), can be a protective factor against DR-related damage [80]. In PRDX4-knockout (PRDX4-KO) mice, STZ-induced hyperglycemia resulted in significant retinal neurodegeneration, evidenced by reduced retinal thickness, increased apoptosis in the ganglion cell layer, elevated GFAP, and decreased glutamine synthetase. Additionally, PRDX4 deficiency exacerbated ER stress and mitochondrial dysfunction, as indicated by elevated levels of glucose-regulated protein 78, activating transcription factor 4, C/EBP homologous protein (CHOP), phosphorylated protein kinase RNA-like endoplasmic reticulum kinase (PERK), swollen mitochondria with disrupted cristae, reduced mitochondrial membrane potential, and lower ATP contents. Oxidative stress was intensified in PRDX4-KO diabetic mice demonstrated by increased dihydroethidium and 8-hydroxy-2′-deoxyguanosine staining. In vitro silencing PRDX4 in Müller cells under hyperglycemic conditions amplified reactive gliosis, apoptosis, ER stress, and oxidative stress, whereas overexpression of PRDX4 mitigated these effects. These findings suggest that enhancing PRDX4 could be a promising therapeutic strategy for DR.

Poly (ADP-ribose) polymerase-1 (PARP-1), a DNA-dependent nuclear enzyme, plays a critical role in the pathophysiology of DR by driving inflammation, oxidative stress, and neovascularization. PARP-1 acts as a downstream effector in both oxidative and nitrosative stress pathways, contributing to endothelial cell proliferation under hypoxic conditions. Under normal conditions, PARP-1 knockout (KO) mice do not show significant phenotypic alterations [81,82]. However, in diabetes, elevated glucose levels upregulate PARP-1 expression, exacerbating vascular complications and inflammation [83]. Consequently, PARP-1 inhibitors, such as nicotinamide and 3-aminobenzamide, have been used to treat STZ-induced diabetic rat models, leading to a significant reduction in angiostatin isoforms, a key regulator of angiogenesis [84,85]. However, PARP-1 inhibitors can exhibit substantial cytotoxic effects at higher doses, requiring careful dose management [86]. Furthermore, while PARP-1 inhibitors effectively reduce inflammation and oxidative stress, their limited impact on vascular complications and neuroprotection necessitates alternative therapeutic approaches. To overcome these challenges, combining PARP-1 inhibitors with other treatments—such as anti-VEGF agents, Rho-associated protein kinase inhibitors, or neuroprotective peptides—has emerged as a promising strategy. By simultaneously targeting multiple disease mechanisms, these combination therapies offer a more comprehensive and effective approach to treating DR.

## 5. Conclusions

DR remains a leading cause of vision loss and blindness among patients with diabetes mellitus. Although several treatments are available, effectively managing the disease continues to present significant challenges. While anti-VEGF therapies and laser treatments offer clinical benefits, limitations such as treatment resistance, side effects, and the need for frequent interventions underscore the urgent need for novel approaches. The molecular mechanisms underlying DR are highly complex and interrelated, making it difficult to develop targeted therapies. Additionally, key biomarkers remain underexplored, limiting early diagnosis and intervention. Given these challenges, emerging therapeutic strategies—including small molecule inhibitors targeting novel molecular pathways, gene-based therapies, and anti-inflammatory agents—show promise in improving patient outcomes. Furthermore, dietary interventions and combination therapies may provide additional benefits by addressing underlying mechanisms such as inflammation and oxidative stress. Repurposing existing drugs for DR treatment also presents a cost-effective strategy that could accelerate the development of new therapeutic options. Advancing early diagnostic tools and integrating these emerging therapeutic strategies could significantly transform DR management, ultimately reducing the burden of vision loss among patients with diabetes mellitus.

## Figures and Tables

**Table 1:** Approved drugs for DR treatment.

Type	Drug Name	Target of Action	Administration Route
Biologics	Bevacizumab	Anti-VEGF	Intravitreal injection
	Ranibizumab		
	Alfibercept		
	Faricimab		
	Brolucizumab		
Small molecules	Conbercept	Glucocorticoid receptor agonist	Intravitreal implant
	Difluprednate		Ophthalmic
	Dexamethason		Intravitreal implant
	Triamcinolone acetonide		Intravitreal injection
	Finerenone	Mineralocorticoid receptor antagonist	Oral
	Fenofibrate	Lipoprotein lipase stimulator (PPAR alpha agonist)	Oral
	Ocriplasmin	Alpha-2 antiplasmin inhibitor, collagen antagonist, fibronectin inhibitor, laminin antagonist plasmin stimulator	Intravitreal injection
	Ripasudil hydrochloride	Rho-associated protein kinase inhibitor	Ophthalmic
	Bromfenac sodium	Cyclooxygenase inhibitor	Ophthalmic
	Nepafenac		

## Data Availability

No new data were created or analyzed in this study.
